# Immunotoxins and Other Conjugates Containing Saporin-S6 for Cancer Therapy

**DOI:** 10.3390/toxins3060697

**Published:** 2011-06-22

**Authors:** Letizia Polito, Massimo Bortolotti, Manuela Pedrazzi, Andrea Bolognesi

**Affiliations:** Department of Experimental Pathology, “Alma Mater Studiorum” University of Bologna, via San Giacomo 14, 40126-Bologna, Italy; Email: letizia.polito@unibo.it (L.P.); massimo.bortolotti2@unibo.it (M.B.); manuela.pedrazzi2@unibo.it (M.P.)

**Keywords:** saporin-S6, plant toxins, immunotoxins, immunoconjugates, immunotherapy, anti-cancer therapy

## Abstract

Ribosome-inactivating proteins (RIPs) are a family of plant toxins that permanently damage ribosomes and possibly other cellular substrates, thus causing cell death. RIPs are mostly divided in two types: Type 1 RIPs that are single-chain enzymatic proteins, and type 2 RIPs that consist of an active A chain (similar to a type 1 RIP) linked to a B chain with lectin properties. RIP-containing conjugates have been used in many experimental strategies against cancer cells, often showing great efficacy in clinical trials. Saporin-S6, a type 1 RIP extracted from *Saponaria officinalis* L. seeds, has been extensively utilized to construct anti-cancer conjugates because of its high enzymatic activity, stability and resistance to conjugation procedures, resulting in the efficient killing of target cells. This review summarizes saporin-S6-containing conjugates and their application in cancer therapy, considering *in-vitro* and *in-vivo* studies both in animal models and in clinical trials. The review is structured on the basis of the targeting of hematological *versus* solid tumors and on the antigen recognized on the cell surface.

## 1. Introduction

Ribosome-inactivating proteins (RIPs) are plant enzymes that damage ribosomes, and possibly other substrates, in an irreversible manner. RIPs are mainly divided into type 1, which consist of a single-chain protein, and type 2, which are composed of an enzymatic A-chain linked to a B-chain possessing lectin properties [1]. The interest in RIPs derives mostly from potential applications after being linked to appropriate carriers, such as monoclonal antibodies (mAbs) or other molecules, to obtain conjugates that are specifically toxic to target cells [2]. These conjugates have been included in experimental therapies against various malignancies, often achieving promising results. Among type 1 RIPs, Saporin-S6 (SAP; from seeds of *Saponaria officinalis *L. [3]) has been widely used to construct anti-cancer conjugates because of its high enzymatic activity [4] that has been related to the negative electrostatic surface potential of the active site pocket [5]. Further positive aspects of SAP are the maintenance of its enzymatic activity after conjugation procedures [6] and its resistance to proteolytic degradation, thus producing very efficient conjugates for the killing of target cells [7].

This review summarizes the applications of SAP-containing conjugates in experimental therapies against hematological and solid tumors, considering *in-vitro* and *in-vivo* studies both in animal models and in clinical trials. Conjugates containing antibodies or their fragments are referred to as immunotoxins (ITs), whereas conjugates having other carriers are denoted as “conjugates”. Unless otherwise specified, the conjugates and the ITs listed in this review have been obtained by chemical conjugation.

## 2. ITs Targeting Hematological Cells

Hematological cells have been extensively studied and targeted with ITs because (i) they have well-known and well-characterized surface molecules against which a panel of mAbs is available; (ii) many surface antigens modulate and are effectively internalized after binding with specific Abs; (iii) fresh cells may be easily tested for IT activity; and (iv) hematological neoplastic cells are easier to access and target *in vivo* compared to solid tumor cells. The main results obtained *in vitro* and in animal models are summarized in Table 1. Clinical trials are reported in Table 2.

**Table 1:** *In vitro* and *in vivo* studies with Saporin-S6 (SAP) containing immunotoxins (ITs) targeting hematological tumors.

			Antitumor Activity			
Antibody	Target Antigen	Tumor	*In vitro *IC_50_ (M)	*In vivo*		Ref.
				Schedule	Effects	
Various	CD2	T-CLL	10^−13^~10^−11^	n.d.	n.d.	[8]
OKT11 7A10C9	CD2	T-CLL	<5 × 10^−13^	n.d.	n.d.	[9]
OKT1	CD5	T-lymphocytes B-CLL	3.2 × 10^−10^; 4~6.8 × 10^−9^	n.d.	n.d.	[10,11]
UCHT1	CD3	lymphocytes	2.1 × 10^−10^	n.d.	n.d.	[6,12]
HB2 × DB7-18	CD7	T-ALL	2.3 × 10^−10^	n.d.	n.d.	[13,14]
HB2	CD7	T-ALL	4.5 × 10^−12^	SCID-HSB2 mice; 1 × 0.5 mg/kg	50% survival at 150 days	[15,16]
BU12	CD19	B-LL		SCID-NALM-6 mice; 3 × 10 μg	40% survival at 110 days	[17]
		BL		SCID-Ramos mice; 1 × 10 μg IT + 10 μg Rituximab	100% survival at 120 days	[18,19]
Rituximab	CD20	NHL	1~3 × 10^−10^	n.d.	n.d.	[20]
F(ab’)_2_ BsAbs	CD22	BL	1.5~6 × 10^−10^	n.d.	n.d.	[21]
OM124	CD22	B lymphoblastoid, BL	<5 × 10^−15^~2.0 × 10^−11^	SCID-Daudi mice 3 × 0.5 mg/kg 2 × 0.5 mg/Kg IT + 60 mg/Kg cyclophosphamide	33% tumor free 66% tumor free	[22]
Ber-H2	CD30	HD	5 × 10^−12^~5 × 10^−14^	n.d.	n.d.	[23]
		ALCL		SCID-JB6 mice 3 × 11.3 μg	CR 80% CR 30%	[24]
		ALCL		SCID-D430B mice 1 × 0.1 mg/kg	CR 66% PR 33%	[25]
IB4	CD38	NHL	2~13 × 10^−12^	n.d.	n.d.	[26]
B7-24	CD80	BL, HD	<10^−11^	n.d.	n.d.	[27]
M24 + IG10	CD80/ CD86	BL, HD	0.3~5.8 × 10^−12^	n.d.	n.d.	[28]
83	CD3/ CD28	lymphocytes	8 × 10^−11^ AC_50_ 2 × 10^−12^	n.d.	n.d.	[29]
		ALL	AC_50_ 10^−11^	n.d.	n.d.	[30]
ATG	various	lymphoma and leukemia cells	5 × 10^−11^~10^−10^	n.d.	n.d.	[31]

ALCL: anaplastic large-cell lymphoma; ALL: acute lymphoblastic leukemia; B-CLL; B-chronic lymphocytic leukemia; BL: Burkitt’s lymphoma; B-LL: B-cell lymphoblastic leukemia; CR: complete remission; HD: Hodgkin’s disease; NHL: non-Hodgkin’s lymphoma; PR: partial remission; n.d.: not determined; T-ALL: T-cell acute lymphoblastic leukemia; T-CLL: T-cell chronic lymphocytic lymphoma.

**Table 2:** Clinical trials in patients with SAP containing ITs.

Antibody	Antigen	Disease	Total Dose	PR	SD/MR	No. patients	Ref.
F(ab’)_2_ BsAb	CD22	NHL	5 mg	-	1	1	[32]
4KB128 + HD6	CD22	B-cell lymphoma	5–20 mg	-	4	4	[33]
F(ab’)_2_ BsAb	CD22	NHL	5–20 mg	-	5	5	[34]
Ber-H2	CD30	HD	0.8 mg/kg	3 (75%)	1 (25%)	4	[35]
Ber-H2	CD30	HD	0.2–0.8 mg/kg	5 (40%)	3 (25%)	12	[36]

HD: Hodgkin’s disease; NHL: non-Hodgkin’s lymphoma; PR: partial remission; SD/MR: stable disease/minor response.

### 2.1. ITs Targeting CD2, CD3 and CD5

SAP was used to prepare several ITs with mAbs against CD2, CD3 and CD5. CD2 is a 50 kDa glycoprotein expressed on the surface of T cells, natural killer (NK) cells, granulocytes and on subsets of monocytes and B cells. Anti-CD2 ITs have been studied as therapies for CD2^+^ lymphomas and leukemias. Various anti-CD2 mAbs (GT2, OKT11, 8E5B3, 8G5B12, 7A10C9) were used by Tazzari *et al*. [8] to produce SAP-containing ITs. These conjugates were found to inhibit protein synthesis in a neoplastic CD2^+^ cell line (SKW-3) and in an IL-2-dependent CD2^+^ lymphoid cell culture (T lymphoblasts) with concentrations causing 50% of protein synthesis inhibition (IC_50_s) ranging from 10^−13^ to 10^−11^ M. The most effective ITs, OKT11- and 7A10C9-SAP, were further investigated in D430B cells (anaplastic large-cell lymphoma), with IC_50_s lower than 5 × 10^−13^ M. Bone marrow purging of contaminating lymphoma cells by means of CD34^+^ cell purification and subsequent OKT11-SAP treatment resulted in >5 logs of lymphoma cell killing without any significant reduction in stem cell clonogenic properties [9].

CD5 (gp67) is a developmental marker of T lymphocytes that is also present on the surface of B cell subsets. Anti-CD5 ITs have been devised as a tool for the therapy of T-cell lymphomas and leukemias and CD5^+^ B-chronic lymphocytic leukemia (B-CLL). The OKT1-SAP IT showed very fast kinetics. Indeed, T-lymphocyte killing was achieved with only 5 min of exposure to the IT. The concentration causing 50% of inhibition of T-lymphocyte DNA synthesis was 3.2 × 10^−10^ M. This IT was given as a single intravenous (i.v.) injection at non-toxic dosages (0.16–1.3 mg/kg) in cynomolgus monkeys. An initial rapid decline in plasma concentration (t_1/2_α = 1.0–4.1 h) was followed by a long-lasting slower decrease (t_1/2_β = 11.6–20.6 h) [10]. This IT was also tested on fresh B-CLL cells from 31 patients. In 90% of cases, OKT1-SAP specifically suppressed B-CLL cell proliferation in a dose-related manner, with IC_50_s 4.0–6.8 × 10^−9^ M [11].

In a comparative study, different CD antigens were targeted by specific mAbs [OKT11 (anti-CD2), SOT3 (anti-CD3) and SOT1a (anti-CD5)] linked to SAP. The anti-CD2 IT OKT11-SAP was 10-fold less effective than the other two ITs. However, OKT11-SAP, similarly to SOT3- and SOT1a-containing ITs, was able to accomplish T lymphocyte killing after less than 10 min of exposure in the absence of adjuvant molecules [37].

An IT containing the anti-CD3 mAb UCHT1 linked to SAP showed cytotoxic effects on stimulated human peripheral lymphocytes. The IT showed an IC_50_ of 2.1 × 10^−10^ M with a 2 log increase in SAP toxicity [6,12].

### 2.2. ITs Targeting CD7

CD7 is a 40 kDa determinant expressed by most T-cell acute lymphoblastic leukemias (T-ALLs) and the majority of normal mature peripheral T cells. HB2 has been one of the most used mAbs to produce anti-CD7 ITs. SAP was effectively delivered to the T-ALL cell line HSB-2 by a F(ab’γ)_2_ bispecific antibody (BsAb) specific for SAP and CD7 antigen. The IT showed an IC_50 _of 2.3 × 10^−10^ M and a rate of protein synthesis inactivation very similar to that described for conventional ITs (time taken for a one log inhibition of protein synthesis compared with controls (t_10_) of 46 h at 10^−9^ M and 226 h at 10^−10^ M) [13]. The cytotoxic performance of an HB2-derived BsAb, also reacting with SAP (HB2 × DB7-18), was investigated *in vitro*. With HSB-2 cells the HB2 × DB7-18 BsAb had an IC_50_ of 2.3 × 10^−10^ M with a 435-fold increase in SAP toxicity [14]. Comparative experiments showed that the HB2-SAP IT was six times more effective than HB2 × DB7-18 and 98 times more effective than the corresponding quadroma BsAb Q1.1 [38]. An HB2-SAP IT was found to specifically inhibit HSB-2 cell line protein synthesis with an IC_50_ of 4.5 × 10^−12^ M and it significantly prolonged the survival of several combined immunodeficient (SCID) mice injected with HSB-2 cells. This therapeutic effect was seen with a single injection of 10 µg (0.5 mg/kg) of IT given at day +7 [15], and it was even more evident when the IT was administered as three daily injections of 10 µg on days +7, +8 and +9 [16]. Further experiments revealed that host-mediated antibody dependent cell cytotoxicity (ADCC) contributes to the *in vivo* therapeutic efficacy of HB2-SAP IT, as demonstrated by both the reduced activity of an IT constructed with the HB2 F(ab’)_2_ fragment, which is incapable of recruiting NK cells [39], and the reduced activity of HB2-SAP in NOD/SCID mice, which have reduced cytolytic NK activity [40]. *In vivo* assessments of the same IT constructed with either a hindered (HB2-SMPT-SAP) or non-hindered (HB2-SPDP-SAP) disulphide bond [41], and containing one or two SAP moieties [42], failed to reveal significant differences in pharmacokinetic [41] or therapeutic effects [41,42].

### 2.3. ITs Targeting CD19

CD19 is a 95 kDa glycoprotein that functions as a response regulator that modulates B-cell differentiation. It is expressed on the B lymphocyte lineage from the beginning of B-cell commitment to plasma cell differentiation, and it is also present on B-cell lymphomas and leukemias. HD37 mAb conjugated to SAP is an IT found to kill more than 2 logs of clonogenic B-CLL cells from patients after a 2 h incubation at a concentration not toxic to non-target cells [43].

The BU12-SAP IT was constructed by covalent coupling of SAP to the BU12 mAb. This IT is selectively cytotoxic *in vitro* in a dose-dependent manner for the CD19^+^ B-cell acute lymphoblastic leukemia cell line NALM-6, but it exhibits no toxicity for the CD19^−^ T-ALL cell line HSB-2. The survival of SCID mice challenged with NALM-6 cells was significantly prolonged compared with sham-treated control animals by a course of therapy with 3 × 10 μg doses of BU12-SAP but not with an irrelevant anti-CD7 IT [17]. Similar results were obtained with SCID mice challenged with the CD19^+ ^human Burkitt’s lymphoma cell line Ramos treated with 3 doses of BU12-SAP IT starting at day + 7 from tumor cell injection [18]. Flavell *et al.* explored the augmentative effect of Rituximab on BU12-SAP in a model of human lymphoma. A combination of 10 μg Rituximab + 10 μg BU12-SAP completely abolished Ramos cell proliferation *in vitro* and induced a significantly greater degree of apoptosis. In SCID-Ramos mice, treatment with a mixture of 10 μg Rituximab + 10 μg BU12-SAP starting at day +7 from i.v. injection of tumor cells had a better therapeutic effect than the individual agents. Indeed, the IT used individually significantly prolonged survival (maximal survival time from 35 to 75 days), but all animals succumbed by day 75. When the IT and Rituximab were used in combination, all animals survived and were disease free at day +120. The therapeutic efficacy was reduced in SCID-Ramos mice depleted of serum complement, whereas NK cell depletion failed to show any convincing role for ADCC [19].

### 2.4. ITs Targeting CD20

The CD20 antigen, a 33–37 kDa membrane protein of unknown function, is an excellent immunotherapeutic target as it is expressed only on mature B cells and not on B-cell precursors. The chimaeric mAb Rituximab has emerged as an effective single agent for the treatment of patients with CD20^+^ non-Hodgkin’s lymphoma (NHL) or chronic lymphocytic leukemia (CLL). In 1997, Rituximab was approved by the US FDA for the treatment of recurrent/refractory follicular NHL and of untreated aggressive NHL in combination with the cyclophosphamide-hydroxydaunorubicin-oncovin-prednisone (CHOP) regimen. Rituximab treatment showed a response rate of about 50% in relapsed low-grade NHL [44]. A Rituximab-SAP IT was constructed and tested *in vitro* for its anticancer properties. Upon conjugation, the toxicity of SAP for target cells increased by about 3 logs, with IC_50_ values of 1–3 × 10^−10^ M. The percentage of AnnexinV^+^ cells was over 95% in cell lines treated with 10^−8^ M IT. The complete elimination of Raji clones was achieved with 10^−8^ M IT, whereas a mixture of free RIP and mAb gave about 90% clonogenic growth. Rituximab-SAP was also found to induce apoptosis in 80% of lymphoma cells from NHL patients. Moreover, Raji sensitivity to the IT was augmented when cells were coincubated with Fludarabine, one of the most widely used chemotherapy drugs. The synergistic toxic effect of these two drugs led to the total elimination of the neoplastic population [20].

### 2.5. ITs Targeting CD22

CD22 is a 135 kDa B-cell restricted sialoglycoprotein that has a role as a component of the B-cell activation complex and as an adhesion molecule. CD22 is expressed on the surface of B-cells only at mature stages of differentiation, and it is also expressed by the majority of B-cell malignancies. SAP was found to be effectively delivered to malignant B-cells by four different anti-CD22/anti-SAP F(ab’)_2_ BsAbs. Each α-CD22 BsAb increased SAP toxicity up to 1000-fold, reaching IC_50_ values of 1.5–6.0 × 10^−10^ M for both Daudi and Raji target cells. Pairs of anti-CD22 BsAbs that recognize different non-blocking epitopes on the SAP molecule are able to bind SAP more tightly to the target cell and, as a consequence, increase SAP cytotoxicity and lower the IC_50_ to 2 × 10^−11^ M [21].

In a clinical trial, F(ab’)_2_ BsAb was used to deliver SAP in the treatment of one patient with end-stage NHL. Giving 5 mg of SAP complexed with a pair (50 mg) of anti-CD22 BsAbs over 15 days provided a marked clinical response, including complete clearance of tumor from the blood, clearance of ascites and shrinkage of tumor masses. The patient developed a strong anti-mouse Fab response 28 days after the start of treatment. No anti-SAP response was detected [32]. Four patients were treated with two BsAbs directed to different epitopes of CD22 (4KB128 and HD6) and SAP as therapy for low grade end-stage B-cell lymphoma. The patients received between 3 and 6 doses of IT at weekly intervals with infusions containing escalating doses of the complexes (ranging between 1 and 4 mg) given every hour. Toxic effects were minimal (grade I), with mild fever, weakness and myalgia for 1–2 days after the treatment. One patient had an antibody response to mouse Fab’ and SAP. All patients showed rapid and beneficial responses to the treatment. In the week after their last treatment, all showed at least a 50% reduction in measurable disease, although no response persisted for the 28 days defined as being necessary for a partial response. Circulating tumor cells were cleared in the three patients with blood involvement. Ascitic fluid and pleural effusions disappeared. Lymph nodes showed reductions in size of at least 50% in three patients. One patient had complete resolution of severe neutropenia and one lost his transfusion requirement [33]. French *et al*. treated five low grade NHL patients with a cocktail of two BsAbs, which bind cooperatively to SAP, and CD22 on B cells. Patients were treated with weekly doses of the IT (3 or 6 infusions between 2 and 4 mg) for a period of up to 6 weeks. All weekly treatments were well tolerated and all five patients showed marked clinical responses, with removal of the bulk of their disease following treatment. The tumor was removed from circulation (4/5), ascitic fluid and pleural effusions were eradicated (2/2), splenomegaly (1/1) and enlarged lymph nodes (5/5) were shrunk, and marrow was partially cleaned, resulting in significant improvements in function in some patients. Most of these beneficial effects were achieved rapidly, after just three treatments, but in all cases, the duration of the response was short (2–4 weeks) [34].

SAP has also been linked to the α-CD22 mAb OM124. This IT is probably the most effective at inhibiting protein synthesis in various CD22^+^ target cell lines (Daudi, EHM, BJAB, Raji and BM21), with IC_50_s ranging from <5 × 10^−15^ (on EHM cells) to 2 × 10^−11^, and IC_90_s ranging from 5 × 10^−14^ to 10^−10^ M. Treatment with the IT (0.5 mg/kg at days +1, +4 and +7 after tumor challenge) was found to significantly extend the survival time of SCID mice bearing transplanted Daudi cells, with 33% of all animals tumor-free after 220 days. The combination of cyclophosphamide (60 mg/kg at days +1 and +2) and OM124-SAP (as above) augmented the efficacy so that 66% of the animals remained tumor-free after 220 days [22]. The humanized anti-CD22 mAb Epratuzumab conjugated to SAP is specifically cytotoxic to CD22^+^ lymphoma cell lines, being able to completely eliminate target cells while sparing non-target cells. Its cytotoxic effect was demonstrated *in vitro* by the complete loss of viability, and this was found to be related to the complete inhibition of protein synthesis and strong induction of apoptosis. This IT completely abolished the clonogenic growth of both BJAB and Raji cells [45].

### 2.6. ITs Targeting CD30

CD30 is a member of the TNF receptor family. It was originally described as a marker of Hodgkin and Reed-Sternberg cells in Hodgkin’s lymphoma. CD30 expression is mostly restricted to virus-infected lymphocytes, neoplasms of lymphoid origin and a subset of activated T cells that produce Th2-type cytokines. The mAb Ber-H2 was conjugated with SAP giving an IT that specifically inhibits protein synthesis in Hodgkin-derived cell lines with IC_50_s ranging from 5 × 10^−12 ^to 5 × 10^−14^ M [46]. Because of transient hepatotoxicity observed in patients treated with Ber-H2-SAP, its accumulation in rat liver cells was studied. Adherent cultured non-parenchymal cells, mostly Kupffer cells, were found to accumulate this IT approximately 10 times more than parenchymal cells and to be 100-fold more sensitive to it [23]. In a SCID mouse model of human xenografted CD30^+^ anaplastic large-cell lymphoma (ALCL) (the JB6 cell line), a 3-day treatment with non-toxic doses of Ber-H2-SAP (50% of LD_50_) induced complete remission in 80% of mice when treatment started 24 h after tumor transplantation. In contrast, injection of the IT at later stages of tumor growth (mice bearing subcutaneous tumors of 40- to 60-mm^3^ volume) significantly delayed tumor growth rate but induced complete remission in only 30% of mice. Thus, the efficacy of treatment with this IT appears to be related to tumor size [24]. Strong anti-tumor activity for Ber-H2-SAP was also observed in another SCID mouse model of ALCL (the D430B cell line). A dose of 0.1 mg/kg IT given 48 h after tumor transplantation caused complete remission in 4/6 mice and partial remission in 2/6 mice [25]. Ber-H2-SAP was studied in a phase I/II clinical trial in four patients with advanced, refractory Hodgkin’s disease. Injection of this IT at a dose of 0.8 mg/kg (administered as two injections of 0.4 or as a single injection of 0.8) had an anti-tumor effect with only mild and transient hepatotoxicity. In all patients there was a rapid (7 days after the treatment) reduction in tumor mass, and 3/4 patients achieved partial remission. Two patients displayed complete relief of systemic symptoms. The responses lasted 6–10 weeks; there was no growth at new sites, but regrowth occurred at all the original sites except for the skin of one patient [35]. This study was extended to 12 patients with advanced Hodgkin’s disease (HD). A single i.v. injection of Ber-H2-SAP at doses 0.2–0.8 mg/kg induced a rapid reduction in tumor mass in about 60% of patients with partial remission in 5/12 patients and MR in 2/12. This study is the first example of immunotherapy for HD and is the first case of cancer therapy with a type 1 RIP-containing IT leading to remission [36].

### 2.7. ITs Targeting CD38

CD38 is strongly expressed by myeloma, lymphoma and leukemia cells. A limitation to the potential *in vivo* use of an anti-CD38 IT is the rather wide expression of CD38 antigen by several cell types. In a model of Ramos cells growing aggressively in SCID mice, an anti-CD38 IT (OKT10-SAP) was found to significantly prolong survival as compared with controls; but all the treated animals eventually succumbed to the disease [47].

The anti-CD38 IT IB4-SAP was studied *in vitro* in CD38^+^ human cell lines (Raji, HBL6, L540 and CEM) and in CD38^+^ neoplastic cells from a NHL patient. HBL6, L540 and Raji cells were highly sensitive to the IT with IC_50_ values ranging from 2 to 13 × 10^−12^ M and concentration causing 100% of protein synthesis inhibition (IC_100_) at 10^−10^ M. CD38^+^ neoplastic cells obtained from a NHL patient were completely eliminated with 10^−8^ M IT. Colony-forming unit cell (CFU-c) rescue by bone marrow precursors was maintained after exposure to the IT [26].

### 2.8. ITs Targeting Costimulatory Antigens

The most important regulatory signal for T-cell activation is the interaction between the antigen presenting cell (APC) surface molecules CD80 (B7-1) and CD86 (B7-2) and the T-cell molecules CD28 and cytotoxic T-lymphocyte antigen-4 (CTLA-4). These molecules are also present on the surface of many tumor cells and their expression at high levels often correlates with a poor prognosis.

#### 2.8.1. ITs Targeting CD80/CD86

Strong expression of the CD80 antigen has been described on most Hodgkin and Reed-Sternberg cells independently of their histological subtype, whereas the CD86 antigen is particularly expressed by the histological subtypes showing mixed cellularity or nodular sclerosis. An anti-CD80 IT, B7-24-SAP, exhibits strong cytotoxicity against the CD80^+^ B-cell line Raji and the Reed-Sternberg cell lines HDLM2 and KM/H2 (IC_50_s < 10^−11^ M). In clonogenic assays with Raji cells or KM/H2 cells, 3 or 4 logs of killing, respectively, was observed. No cytotoxicity was found against B7-1^−^ epithelial or endothelial cell lines or against haematopoietic progenitor cells [27].

The mAbs, M24 (anti-CD80) and 1G10 (anti-CD86), after conjugation to SAP, showed specific cytotoxicity for the CD80/CD86-expressing cell lines Raji and L428. These ITs inhibited protein synthesis by target cells with IC_50_s ranging from 0.3 to 5.8 × 10^−12^ M. The anti-CD80 IT appeared to be 1 log more toxic to target cells than the anti-CD86 IT. These ITs at concentrations ≥ 10^−9^ M showed some toxicity to CFU-c. However, the long term colture initiating cells (LTC-IC) assay did not detect any toxicity of these ITs even at the highest concentration tested (10^−7^ M) [28].

#### 2.8.2. ITs Targeting CD156 (CTLA-4)

The CTLA-4 (CD152) co-stimulatory molecule, a homologue of CD28, plays an inhibitory role in regulating T cell responses by interacting with CD80/CD86 on APCs. CTLA-4 expression is induced on T cells upon activation, which is followed by its rapid internalization, and its membrane expression is restricted to activated T cells. ITs containing recombinant human-derived anti-CTLA-4 single-chain Fv fragments (scFv) (namely scFv-83 and -40) linked to SAP induce apoptosis in activated T lymphocytes. These ITs were able to specifically inhibit the mixed lymphocyte reaction between T-lymphocytes and dendritic cells, and that between T lymphocytes and an Epstein-Barr virus (EBV) positive lymphoblastoid B cell line. The most effective IT (83-SAP) tested on CD3/CD28-stimulated lymphocytes showed an IC_50_ of 8 × 10^−11 ^M and a concentration causing apoptosis in 50% of cells (AC_50_) of 2 × 10^−12 ^M [29,48]. No toxicity for hematopoietic precursors was reported. 83-SAP was tested in a model of tumor rejection consisting of C57BL/6 mice bearing a murine H.end endothelioma cell line derived from DBA/2 mice. The IT was injected at days 0, 1 and 2 at the dose of 4 μg. Lymphoid infiltration due to the presence of the tumor was markedly reduced, demonstrating that the IT was actually available and active *in vivo* [29]. Furthermore, 83-SAP at 10^−8^ M causes apoptosis in more than 90% of neoplastic cells from an acute myeloid leukemia and its AC_50_ is in the 10^−11^ M range [30].

### 2.9. ITs Containing Polyclonal Antibodies

Despite their high selectivity, mAbs are frequently unable to mediate the killing of all the malignant cells. Indeed, differential surface marker expression and variable cell surface phenotypes can be observed in patients with the same type of hematological neoplasia. Polyclonal antibodies, which bind several antigens on a wide range of cells, offer the great advantage of preventing the escape of neoplastic clones during immunotherapy. Anti-thymocyte globulins (ATG) consist of a mixture of polyclonal antibodies that are specific for cell surface proteins, which include CD2, CD3, CD4, CD5, CD7, CD8, CD11a, CD19, CD20, CD25, CD28 and many others. ATG-SAP IT has a strong effect on lymphoma- and leukaemia-derived cell lines, reducing protein synthesis by 2–3 logs (IC_50_ from 5.3 × 10^−11^ to 1.3 × 10^−10^ M) with respect to unconjugated SAP (IC_50_ from 5.9 × 10^−9^ to 3.2 × 10^−8^ M). This IT causes cell killing without the need for complement dependent cytotoxicity or ADCC [31]. 

### 2.10. Cocktails of Various Anti-Lymphocyte ITs

Anti-CD7 and anti-CD38 F(ab’γ)_2_ BsAbs were used, alone or in combination, to deliver SAP to the cell surface of two human T-ALL cell lines (HSB-2 and HPB-ALL). Used alone against HSB-2 cells, the anti-CD7 BsAb HB2 × DB7-18 increased SAP toxicity 435-fold and the anti-CD38 BsAb OKT10 × RabSap increased it 286-fold. When combined, the two BsAbs were 10-fold more effective than the best single BsAb. On HPB-ALL cells, the CD7 BsAb HB2 × DB7-18 increased SAP toxicity only eight-fold, whereas the CD38 BsAb OKT10 × RabSap was highly effective, increasing SAP toxicity 80,000-fold. Interestingly, when both the anti-CD7BsAb HB2 × DB7-18 and anti-CD38 BsAb OKT10 × RabSap were used in combination, they were somewhat less effective than OKT10 × RabSap used alone. Indeed, the combination gave only a 50,000-fold increase in SAP toxicity compared with the 80,000-fold increase obtained with OKT10 × RabSap alone [14].

Treatment of SCID/CEM mice with a single IT containing the parental murine IgG1 anti-CD7 or anti-CD38 led to a delay in the development of leukemia, but 90% of animals treated with either IT developed disseminated leukemia cell growth. The combination of HB2-SAP (anti-CD7) plus OKT10-SAP (anti-CD38) administered in 3 × 10 µg i.v. doses significantly increased survival time and the number of leukemia-free animals (60%) [49]. 

SCID mice challenged with the CD19^+ ^CD38^+^ human Burkitt’s lymphoma cell line Ramos treated with three daily injections of anti-CD19 BU12-SAP IT or of anti-CD38 OKT10-SAP IT starting at day +7 showed significantly prolonged survival. When both ITs were used in combination at equivalent doses, the therapeutic outcome was significantly improved over that obtained with single IT therapy, with 20% of animals surviving disease-free at 300 days [18]. Similar or even better results were achieved in the same Ramos/SCID mouse model with a mixture of three SAP ITs, anti-CD19, anti-CD22 and anti-CD38 [50].

## 3. ITs Targeting Solid Tumor Antigens

Toxin-based conjugates have proven effective against lymphomas and leukemia, but the clinical results for solid tumor treatment have been quite disappointing. The principal reason is that ITs and other conjugates enter into solid tumors poorly and unevenly. Tight junctions between tumor cells, high interstitial pressure and heterogeneous blood supply are the main hurdles to conjugate penetration into the tumor mass. Thus, many strategies involving the use of recombinant antibody fragments, adjuvants (e.g., saponin) and photochemical internalization (PCI) have been investigated to improve the anti-tumor efficacy of conjugates [51]. The main results obtained *in vitro* and in animal models are summarized in Table 3.

**Table 3:** SAP containing ITs or conjugates targeting solid tumors.

			Antitumor Activity			
Carrier	Target Antigen	Tumor	*In vitro *IC_50_ (M)	*In vivo*		Ref.
				Schedule	Effects	
EGF	EGFR	Sarcoma	2.4 × 10^−9 ^w/o saponin 6.7 × 10^−13 ^with saponin	n.d.	n.d.	[52]
		Adenocarcinoma		BALB/c-TSA mice 4 × 280 μg/kg	71% TGR at 20 days	[53]
				6 × 5.6 μg/kg + 1670 μg/kg saponin	94% TGR at 25 days	[54]
		Cervical cancer		SCID-cervical cancer mice6 × 15 μg	50–60% TGR at 30 days	[55]
FGF	FGFR	Melanoma, teratocarcinoma and neuroblastoma	10^−9^~10^−11^	BALB/c-neuroblastoma mice 4 × 0.5 μg/kg	CR 20%	[56]
FGF-2	FGFR	Bladder cancer	1.4 × 10^−8^~ 1.3 × 10^−10^	n.d.	n.d.	[57]
bFGF	FGFR	Prostatic carcinoma		Athymic nude mice-DU1454 × 5 μg/kg	95% TG Rat 38 days	[58]
ch25A11	CDCP1	Prostate carcinoma		SCID CB17 mice 3 × 0.4 mg/kg	66% TGR at 23 days	[59]
hj591	PSMA	Prostate carcinoma	2 × 10^−9^~1.4 × 10^−10^	Athymic nude mice-LNCaP 4 × 32 μg	83–90% TG Rat 49 days	[60]
Ep2	HMW-MAA	Melanoma	10^−10^	n.d.	n.d.	[61]
ML30	HSP65	Leukaemic monocyte lymphoma	10^−9^	n.d.	n.d.	[62]
		Pancreatic carcinoma		SCID-HPC-4 mice 6 × 10^−7^ M	TR 15.9 IT TR 48.7 PBS	
48–127	gp54	Bladder tumor	10^−9^	n.d.	n.d.	[63]
I/F8 scFv	ALCAM/ CD166	Various	2.4~5 × 10^−9^	n.d.	n.d.	[64]
7E4B11	RPTPβ	Astrocytic tumor		Athymic nude mice-glioblastoma 4 × 30 μg	73% TGD	[65]

CR: complete remission; n.d.: not determined; TGR: tumor growth reduction; TGD: tumor growth delay; TR: tumor ratio (tumor volume day 6/tumor volume day 0).

### 3.1. Conjugates Targeting Growth Factor Receptors

It is well known that growth factors play an important role in normal cell proliferation by stimulating growth factor receptors located on the cell surface. Tumor cells express high levels of growth factor receptors that theoretically can serve as therapeutic targets in cancer treatment.

#### 3.1.1. Conjugates Targeting EGFR

The epidermal growth factor receptor (EGFR) is overexpressed in many different types of solid tumors and is associated with metastasis and poor prognosis.

Two of the main problems associated with administering EGFR-targeted toxins in tumor therapy are severe systemic side effects and low transfer of the toxins into the cytosol after binding to the tumor cell surface.

Members of the HER/erbB receptor family seem to be involved in several human cancers. Overexpression of these receptors has been found in many types of epithelial, mesenchymal and neural cancer, and it correlates with tumor progression and with reduced patient survival [66]. Rhabdomyosarcoma cells (RD/18) were treated with an indirect IT consisting of a murine mAb recognizing EGF-R (clone 528) followed by a secondary F(ab’)_2_ antimouse immunoglobulin linked to SAP. This IT induced apoptosis and significantly inhibited cell growth and protein synthesis (IC_50_ 9.5 × 10^−10 ^M) [67].

Recombinant SAP [68] has been linked to EGF both directly (SE) and via an adapter (SA2E). The adapter is designed to improve cytosolic uptake and to retain the toxin inside the cytosol. Heisler *et al*. evaluated the toxicity of SA2E on an EGFR^+^ cell line (HER14 cells derived from mouse embryo NIH-3T3 cells transfected with human EGFR). Pre-incubation with an adjuvant (saponin) enhanced the cytotoxicity of SA2E more than 3 logs (from an IC_50_ of 2.4 × 10^−9^ to 6.7 × 10^−13^ M) [52]. The efficacy with which SE and SAE2 inhibit tumor growth was evaluated in BALB/c mice bearing adenocarcinoma TSA-EGFR^+^ cells. The lethal dose for mice was three times lower for SA2E than for SE. Mice were injected with 1.25 × 10^5^ cells and 5 μg (280 μg/kg) of conjugate was injected s.c. 4 h later in the immediate vicinity (day 0). Further doses were applied on days 3, 6 and 11. The average tumor volume exceeded 5 mm^3 ^on days 10, 11, 14 and 18 for SAP-treated, untreated, SE-treated and SA2E-treated mice, respectively. After 20 days of treatment, SE only reduced the average weight of induced tumors by 33%, whereas SA2E-treated mice exhibited 71% tumor reduction. Additionally, severe side effects like hyperalgesia, alopecia and death were drastically reduced in SA2E-treated animals [53]. Furthermore, 6 s.c. injections of 30 μg saponin and 0.1 μg SA2E (equivalent to 5.6 μg/kg) reduced the mean tumor volume after 25 days, by a remarkable 94% compared to controls. Although saponin alone had no effect on tumor growth, SA2E alone reduced the mean tumor volume slightly by 42% compared to the controls. Surprisingly, in the i.p. injection saponin/SA2E and SA2E alone did not inhibit tumor growth [54]. In SCID beige mice bearing freshly isolated cervical cancer cells, treatment with 6 s.c. injections of 5 and 10 μg of SA2E decelerated tumor growth by about 20–30%, whereas treatment with 15 μg reduced tumor growth by 50–60% compared to the control group. However, the antitumor effect of SA2E was restricted to the treatment period, and after the SA2E applications were discontinued the tumors showed rapid outgrowth [55].

Weyergang *et al.* demonstrated that a conjugate based on EGF linked to SAP is efficiently endocytosed in two EGFR^+^ cell lines (NuTu-19 from an epithelial ovarian tumor and A-431 from a skin carcinoma). Moreover, the photochemical internalization (PCI) of EGF-SAP improved both the efficacy and specificity of the conjugate. Indeed, PCI enhanced the efficacy of EGF-SAP by about 1000 times in NuTu-19 cells. At a dose where the photochemical treatment reduced viability by approximately 50%, the dose causing death in 95% of population (LD_95_) for PCI of SAP was about 10^−9^ M, whereas the LD_95_ for PCI of the conjugate was about 10^−12^ M. PCI of EGF-SAP was several hundred-fold less efficient at enhancing cytotoxicity in non-target cells than in target cells [69]. PCI has been used to deliver an IT consisting of the anti-EGFR mAb cetuximab linked to SAP into various types of EGFR^+^ carcinoma cell lines (colorectal, prostate, epidermoid). The PCI treatment enhanced the cytotoxicity of the IT in a synergistic manner. Indeed, PCI improved the IT toxicity approximately 10–20 times [70].

#### 3.1.2. Conjugates Targeting FGFR

Fibroblast growth factor receptors (FGFRs) are a family of at least 12 different proteins. Many solid tumors express receptors binding basic FGF (also named FGF-2 or bFGF). A conjugate containing basic FGF linked to SAP was found to be toxic to FGFR^+^ cell lines (human melanoma, teratocarcinoma and neuroblastoma). The conjugate showed IC_50_s ranging from 10^−11^ to 10^−9^ M, whereas unconjugated SAP had IC_50_ values >10^−7 ^M. BALB/c mice bearing human melanoma, teratocarcinoma or neuroblastoma cells were treated with 4 i.v. injections of 0.5 µg/kg conjugate beginning on day +5. Significant tumor inhibition was seen on day 35 for each of the tumor types examined and complete tumor regression was observed in 20% of the neuroblastoma xenografts. No cumulative toxicity was observed [56]. The same conjugate was also evaluated in C75BL/6 mice bearing B16-F10 cells (murine melanoma). The conjugate increased survival time, inhibited tumor growth and decreased metastasis [71].

The chemical and the recombinant FGF-2-SAP conjugates have potent cytotoxicity in malignant bladder cell lines with IC_50_s ranging from 1.3 × 10^−10^ to 1.4 × 10^−8^ M, whereas cells derived from normal fetal bladder were found to be less sensitive to FGF-2-SAP (IC_50_ > 10^−7^ M) [57].

The antitumor activity of recombinant bFGF-SAP was examined in athymic nude mice bearing a prostatic carcinoma cell line (DU145). The conjugate (4 × 5 µg/kg) was administrated by i.v. injection, intraperitoneal injection, or local or distal s.c. injection beginning 5 days after s.c. tumor implantation. No significant difference was observed between the groups receiving the conjugate via i.v. injection *versus* intraperitoneal injection out to day 38. However, tumors receiving local s.c. injections were significantly smaller. Moreover, the conjugate induced dramatic reduction of large (450 mm^3^) established tumors out to 20 days after treatment in 2 animals who received 50 µg/kg bFGF-SAP on day +75 and +85 after tumor injection [58]. 

### 3.2. Conjugates Targeting the Transferrin Receptor

The transferrin receptor (TfR) is expressed on all normal tissue cells, but it is chosen as an immunotherapy target for gliomas and other tumor cells because of the high iron requirement of rapidly proliferating neoplasms. ITs targeting the TfR were hypothesized to possess sufficient specificity to eliminate neoplastic cells in the central nervous system or in other compartments where the delivery of ITs to a tumor does not require transvascular transport. Two different conceptual approaches have been designed to target TfR on cancer cells. Conjugates have been made using the plasma protein transferrin as a carrier, and alternatively, anti-TfR mAbs have been used. Both types of proteins can be used to deliver the RIP to target cells; however, Tf-based conjugates have two major disadvantages with respect to mAb-based conjugates as they are strongly influenced by both the presence of free transferrin and the saturation state of iron [72].

A transferrin-SAP conjugate was found to be internalized via binding with TfR. The conjugate inhibited K562 (human erythroleukaemia) cell proliferation and, in a clonogenic assay, markedly inhibited K562 colony formation (close to 100% at 10^−9^ M) [73]. Conjugation of IgG3-avidin specific for the human-transferrin receptor (anti-hTfR IgG3-Av) with biotinylated SAP was found to enhance the cytotoxic effects of SAP in TfR bearing IM-9 (EBV-transformed lymphoblastoid cell line) and U266 (a lymphoblast cell line) cells. Indeed, protein synthesis was strongly reduced after 48 h of 10^−9^ M IT treatment in both cell lines [74].

Recently, a transferrin-SAP conjugate has been assayed on two glioblastoma multiforme cell lines, GL15 and U87. The conjugate resulted active on both cell lines but after 72 h of incubation with 10^−9^ M transferrin-SAP less than 50% of tumor cells was killed [75].

### 3.3. ITs Targeting Prostatic Antigens

Prostate cancer is a good target for antibody-based therapy because the non-vital function of the prostate allows extending the spectrum of target molecules to tissue-specific markers, not restricted to tumor cells. Moreover, the usually small size metastases can be easily reached and destroyed by ITs.

Ku70/80 protein is expressed in the nucleus of all cells, but its plasma membrane localization is restricted to tumor cell lines of various lineages. Carcinoma cell lines, with varying surface expression of Ku70/80, were pre-treated with 1 × 10^−8^ M of INCA-X IgG, an anti-Ku70/80 antibody, and then incubated with a SAP-anti-human IgG antibody (Hum-ZAP). The inhibition of proliferation induced by INCA-X/Hum-ZAP ranged from very strong (92% on the prostate carcinoma PC-3) to low (30% on the colorectal carcinoma LS174 T), whereas no effect was seen on the Ku70/80-negative breast carcinoma cell line SK-BR-3 [76].

Tomoregulin is a type 1 transmembrane protein with a short cytoplasmic tail. This protein is selectively expressed in the prostate and brain but it is not expressed in other normal tissues. 2H8, an anti-Tomoregulin murine mAb, was coupled to SAP through a mAb-ZAP secondary antibody (goat antimouse IgG antibody linked to SAP) in indirect IT assays. The SAP-based 2H8 IT was found to specifically bind to Tomoregulin-positive cells and induce a 50% reduction of viability at the concentration of 1.6 × 10^−10^ M after 72 h, whereas Tomoregulin-negative cells remained unaffected [77].

CUB domain-containing protein 1 (CDCP1) is widely expressed in tumors, including prostate cancer, and it is recognized by the chimaeric mAb 25A11 (ch25A11). An IT containing SAP coupled to ch25A11 was evaluated *in vivo* in SCID CB17 mice bearing prostate carcinoma cells. The IT (3 × 0.4 mg/kg) administered i.v. inhibited tumor growth by approximately 66% at day 18, 67% at day 22 and 63% at day 23, whereas no inhibition was observed with s.c. injection of the IT at the same dose. However, both i.v. and s.c. IT administration were found to inhibit tumor metastasis, suggesting that although s.c. IT may not have sufficient bioavailability to inhibit primary tumor growth, the inhibition of metastasis may be a direct result of tumor cell killing in the circulation [59].

PSMA is a prostate-specific cell surface marker of prostate cancer. The antitumor activity of the anti-PMSA humanized biotinylated mAb hJ591 linked to streptavidin SAP (SAZAP) was evaluated in prostate derived cell lines: LNCaP, CWR22Rv1 (both PMSA^+^) and PC-3 (PMSA^−^). The IC_50 _of this IT was 1.4 × 10^−10^ M, 2 × 10^−9^ M and more than 10^−7^ M in LNCaP, CWR22Rv1 and PC-3 cells, respectively. After 72 h of treatment with this IT, the percentage of apoptotic cells was 60% and 40% in LNCaP and CWR22Rv1 cells, respectively, compared to about 5% in PC-3 cells. The conjugate also had anticancer activity in an LNCaP xenograft model in athymic nude mice. The administration of hJ591-SAZAP (4 × 32 μg doses weekly) was found to inhibit tumor growth in IT-treated mice compared to control groups. Indeed, after 7 weeks of treatment, the tumor volume in untreated mice was 5- to 6-fold greater than in IT-treated mice. In addition, serum PSA (prostate-specific antigen) values were 0.39 ± 1.04 ng/mL and 71 ± 52.85 ng/mL for treated and untreated groups, respectively [60].

### 3.4. ITs Targeting Other Antigens on Solid Cancers

Selective elimination of multidrug resistance (MDR)-positive cells (also called LoVo/Dx colon carcinoma cells) was obtained using the mAb MRK16, which recognizes P-glycoprotein 170, and a sheep anti-mouse immunoglobulin conjugated to SAP. When the IT was used at concentrations of 10^−8^ and 10^−7 ^M, the inhibition of protein synthesis was 73% and 87%, respectively. MRK16 followed by the indirect IT was found to eliminate 99% of MDR cells from a bone marrow suspension [78].

High-molecular-weight melanoma-associated antigen (HMW-MAA) is expressed on a large majority of melanomas but not on most normal or other neoplastic tissues. The mAb Ep2, recognizing an epitope of HMW-MAA, was conjugated to SAP. Ep2-SAP IT efficiently killed antigen expressing cells with an IC_50 _of approximately 10^−10^ M [61].

Heat Shock Proteins (HSP) represents a family of highly conserved molecules that under stress conditions play an important physiological role in folding and unfolding of proteins. Moreover HSPs have been described as tumor-associated antigens; they are constitutively overexpressed in the intracellular compartment and sometimes on the surface of tumor cells. The cytotoxic effect of an anti-HSP65 mAb (ML30) on untreated and heat-treated H9 cells (CD4^+^ lymphoma cell line) was evaluated with an indirect IT (ML30 + anti-mouse IgG antibody linked to SAP). The IT showed marked cytotoxicity against heat-stressed H9 cells at 10^−11^ and 10^−13^ M, whereas normal H9 cells were spared [79]. Proliferation of the U937 cell line (a monocytic cell line that constitutively expresses high levels of membrane HSP65) was evaluated using indirect IT or ML30-SAP direct IT. The inhibition of cell proliferation for both ITs was 100% at 10^−7^ M with an IC_50_ of 10^−9^ M. After 6 treatments with 10^−7 ^M IT in SCID mice bearing HPC-4 cells (human pancreatic carcinoma), the treatment ratio (tumor volume on day 6/tumor volume on day 0) was significantly lower in the IT-treated group than in the control group (15.9 for the IT group; 48.7, 44.8, 43.2 for the PBS, SAP and ML30 groups, respectively) [62].

The 48-127 mAb recognizes a glycoprotein (gp54) expressed on all human bladder tumors and on normal urothelium but not on the luminal surface of normal urothelial umbrella cells. The antigen gp54 is expressed also in non proliferating tumor cells. Thus, ITs specific for this antigen will ensure the killing of resting cells, which are usually spared by conventional chemotherapy. A 48-127-SAP IT was toxic to the T24 target cell line derived from bladder carcinoma, with IC_50_s at nM concentrations after 2 h of incubation followed by 72 h in serum-free RPMI. The inhibition of protein synthesis in T24 cells required 2 h of contact with the IT to ensure optimal binding and endocytosis [63].

Carcinoembryonic antigen-related cell adhesion molecule 6 (CEACAM6) is a glycosylphosphatidylinositol-linked cell surface protein that is overexpressed in a variety of human cancers, including the majority of pancreatic adenocarcinomas. Exposure of pancreatic ductal adenocarcinoma cells (BxPC3) to anti-CEACAM6 antibody (By114) followed by secondary SAP-conjugated immunoglobulin was found to induce marked cytotoxicity. In athymic nude mice bearing BxPC3 cells, the administration of By114 followed by IgG-SAP by two tail vein injections separated by two weeks caused a marked reduction in mean tumor volume after 6 weeks and in some cases tumor regression occurred [80].

Activated leukocyte cell adhesion molecule (ALCAM/CD166) is a member of the immunoglobulin gene superfamily. It is expressed on a number of carcinoma cells and cell lines and in the invasive cells of melanocytic skin lesions where its expression correlates with tumor progression. The toxicity of an IT containing I/F8 scFv linked to SAP was assayed on both human and murine ALCAM/CD166^+^ cell lines, in which it was at least 100-fold higher than that of free SAP with IC_50_s ranging from 2.41 to 5.06 × 10^−9^ M [64].

Receptor protein tyrosine phosphatase β (RPTPβ) is overexpressed primarily in astrocytic tumors. It is known to facilitate tumor cell adhesion and migration through interactions with the extracellular matrix. RPTPβ-7E4B11 and RPTPβ-7A9B5 antibodies were conjugated to SAP and evaluated *in vitro *for their ability to kill glioma cells. These ITs effectively killed tumor cells, reducing their viability by about 50% compared to vehicle-treated cells. The *in vivo* efficacy of 7E4B11 IT was assayed in athymic nude mice bearing glioblastoma cells. At 15 and 30 μg/dose (twice a week for two weeks), the IT produced 25% and 73% tumor growth delays, respectively. All tumors reached the end-point volume by day 44. The median time to end point of PBS-treated mice was 18.6 days, whereas the IT (30 μg/dose) treated mice had a time to end-point of 32.1 days [65]. 

Overexpression of the enzyme human aspartyl (asparaginyl) β-hydroxylase (HAAH) has been detected in a variety of cancers including lung, liver, colon, pancreas, prostate, ovary, bile duct and breast cancer. It has been proposed that upon cellular transformation, HAAH is overexpressed and translocated to the cell surface. The anti HAAH scFv 6–22 was used to deliver goat-anti-human IgG-SAP conjugates (H-Z) into FOCUS cells (a liver tumor cell line) (1 × 10^−8^ M of 6–22 and 2.2 × 10^−8^ M of H-Z). The viability of the 6–22/H-Z treated cells was lower by about 30% after 3 days of incubation [81].

Endosialin/CD248/tumor endothelial marker 1 is expressed in stromal cells, endothelial cells and pericytes in various tumors. The endosialin-positive Ewing’s sarcoma cell line A-673 was exposed to anti-endosialin mAb followed by anti-human IgG conjugated to SAP. The cells were sensitive to the endosialin toxin conjugate with an IC_50_ of approximately 10^−12^ M [82].

The cell cycle-associated overexpression of transcobalamin receptor (CD320) in many cancer cells provides a suitable target for delivering chemotherapeutic drugs and cytotoxic molecules. Various anti-CD320 mAbs were used to deliver IgG-SAP into human colon adenocarcinoma and epidermoid carcinoma cell lines. The IC_50 _was in the 0.625 to 2.5 × 10^−9^ M range for the primary mAb concentration [83].

TEM8 is an integrin-like cell surface protein that is upregulated on tumor blood vessels and it is a potential vascular target for cancer therapy. 293/FlagT8 cells (kidney epithelial cells transformed with adenovirus) are recognized by the anti-TEM8 mAb AF334. Cell viability after treatment with AF334 mAb along with 5 × 10^−9^ M or 1 × 10^−8^ M of SAP-conjugated anti-mouse antibody was almost 40% and 30%, respectively [84].

## 4. Conclusions

The type 1 RIP saporin has been utilized to construct conjugates and ITs against numerous targets with interesting results in many pre-clinical studies (Table 1 and Table 3). Great efficacy has been reported frequently in many models of hematological tumors. In several experiments conducted on mice, treatment with SAP-containing ITs resulted in the elimination of transplanted tumors.

The results achieved in experiments on solid tumors have been less remarkable than those obtained in hematological malignancies, but they have often been better than those reported for other experimental cancer drugs.

Clinical trials with SAP-containing immunotoxins, whose results are summarized in Table 2, show the efficacy of these molecules and their potential for application in lymphoma therapy. Taken together, the studies summarized in this review indicate that saporin-based immunotoxins are almost always extremely powerful *in vitro* and maintain good anti-tumor efficacy *in vivo*. This, at least in part, is due to the intrinsic characteristics of saporin, which is a very stable enzyme. Indeed this protein is very resistant to chemical modifications and conjugation and to blood and endosomal proteases. Moreover, saporin appears to have a wide range of possible intracellular substrates, thus triggering multiple cell death pathways [85]. This multifaceted cytotoxic mechanism renders the selection of RIP-resistant mutants impossible.

Clinical results have demonstrated the efficacy of immunotoxins in cancer patients that are refractory to traditional modalities of treatment: surgery, radiation therapy, and chemotherapy. However so far, the use of immunotoxins in cancer therapy has not completely fulfilled the hopes. Indeed, immunotoxins have multiple problems, mainly: formation of antibodies against the carrier and the RIP, capillary leak syndrome and hepatotoxicity. Modern technologies should permit to circumvent or reduce some of these problems. The use of humanized antibodies can abolish the immune response against the carrier; on the other hand the consequences of the response against the RIP could be easily overcome with cycles of therapy based on the rotation of immunologically different RIPs. Moreover the use of antibody fragments leads to smaller immunotoxins with better penetration in solid tumour tissues than immunotoxins constructed with whole antibodies. Recently many clinical trials have been conducted, and several are ongoing, with immunotoxins, prevalently constructed with deglycosylated A chain of ricin, truncated diphtheria toxin or *Pseudomonas* exotoxin A, but not with saporin. Nevertheless, saporin would have many advantages with respect to these toxins, in terms of stability and efficacy, as described above and thanks to the lacking of the natural immunization often present against bacterial toxins.

Ehrlich’s idea of a “magic bullet” postulated one century ago has not been completely realized to date. Nevertheless, over the last two decades, advances in genetic engineering and in receptor biology have led to many improvements in carriers, creating potential for saporin- and other RIP-containing conjugates to be widely used in cancer therapy.
